# Supplementary material for: Health literacy and its determinants among higher education students in the Alentejo region of southern Portugal—A cross-sectional survey
Source: PLoS One. 2024 Sep 24;19(9):e0309806. doi: 10.1371/journal.pone.0309806 (PMC11421802; doi:10.1371/journal.pone.0309806)
Supplement: Attachment — Submitted filename: Response to reviewer 2.docx [file pone.0309806.s001.docx]

**Response to reviewer 2**

Review 2

Reviewer #2: Detailed Section-by-Section Review of the Manuscript

Dear reviewer,

Manuscript Title: Health literacy and its determinants among higher education students in the Alentejo region of southern Portugal - a cross-sectional survey

Thank you for your detailed and constructive feedback on our manuscript. We have made significant revisions based on your comments:

1. Introduction

• Lack of Contextual Background: The introduction provides general information on health literacy but lacks a detailed context specific to the Alentejo region or higher education students in Portugal.

We appreciate your comment about the need for more specific contextual background in relation to the Alentejo region and higher education students in Portugal. In response, we have expanded the introduction to provide a more detailed context that situates our study within the regional and educational contexts of the Alentejo.

The following information has been added:

“Transitioning from a national perspective to a more focused regional context, the Alentejo faces particular challenges. It has the lowest percentage of tertiary educated and high drop-out rates (29). The Alentejo region in southern of Portugal, which is classified both as a Territorial Level 2 (TL2) and a Nomenclature of Territorial Units for Statistics II (NUTS II) region, covers an area of 31,605 km², about 30% of the national territory. This region has a low population density and is threatened by depopulation due to a decreasing young population, an increasing elderly population and low immigration (30,31). Addressing these inequalities requires promoting health literacy and healthy behaviours from an early age, particularly in rural areas facing demographic challenges (32,33).

The regional focus not only demonstrates the existence of educational and health inequalities but also emphasis the pressing necessity to conduct research and implement solutions at the local level. By elucidating the health literacy levels of higher education students in Alentejo, this research can provide insights into regional inequalities and inform policies aimed at reducing these disparities, ultimately benefiting both individual and community health outcomes.

It is therefore imperative to assess the level of health literacy among higher education students and investigate its association with various determinants. To the best of our knowledge, the current level of health literacy among higher education students in Alentejo is unknown, which makes it challenging to design effective health interventions.”

• Limited Discussion on Previous Research: While there is a mention of health literacy's importance, there is sparse discussion of previous studies directly comparing or contrasting this study's findings with existing data.

We have revised the introduction to include a more comprehensive discussion of previous studies on health literacy, specifically addressing how our findings compare and contrast with existing data in the field.

• Theoretical Framework Missing: The introduction lacks a theoretical framework or model that guides the investigation of health literacy determinants.

A theoretical framework guiding our investigation of the determinants of health literacy has been included in the introduction, providing a clearer basis for our study

The following information has been added:

“In order to gain further insight into the pathways linking health literacy to health behaviours and outcomes, Sørensen et al. (2012) developed the Integrated Model of Health Literacy. This theoretical framework delineates the pathways linking health literacy to health behaviours and outcomes. The model identifies the determinants and factors influencing health literacy levels (the antecedents) and their subsequent health outcomes (the consequences) (1). This theoretical framework, predominant in Europe, describes four core competencies of health literacy: access (the ability to seek, find, and obtain health information), understand (the ability to comprehend accessed health information), appraise (the ability to interpret, filter, judge and evaluate the health information that was assessed) and apply (the ability to communicate and utilise the information to make decision to maintain and improve health). The four competencies, namely accessing, understanding, appraising and applying, can be applied across the three domains of the health continuum, including healthcare, disease prevention and health promotion (1).

An understanding of these competencies, serve to highlight the dynamic and holistic nature of health literacy. This encompasses not only the abilities of individuals, but also the inherent complexity of health systems. This framework facilitates comprehension and improvement of health literacy across heterogeneous populations and healthcare settings. The empowerment of individuals through the acquisition of these skills fosters autonomy and informed decision-making in matters pertaining to health (6) thereby enhancing social participation and well-being. Furthermore, the structural organisation and availability of resources have a significant impact on health literacy levels and individuals' ability to make informed health decisions, which in turn affect personal and community health (7).”

• Insufficient Justification for Study: The rationale for why this study is needed in the Alentejo region is not adequately justified.

We have strengthened the rationale for our study in the Alentejo region by highlighting specific gaps in the current literature and emphasising the unique regional challenges that justify our research focus.

The following information has been added:

“It is therefore imperative to assess the level of health literacy among higher education students and investigate its association with various determinants. To the best of our knowledge, the current level of health literacy among higher education students in Alentejo is unknown, which makes it challenging to design effective health interventions. “

• Lack of Hypotheses: No hypotheses are presented, which makes it challenging to understand the expected outcomes or the direction of the research.

We included the following research questions:

- “What is the level of health literacy among higher education students in the Alentejo region of southern Portugal?
- What is the relationship between health literacy and determinants such as socio-demographic variables, the presence of chronic disease, perceived health status, and perceived availability of money for expenses among higher education students in the Alentejo region?

The responses to these questions will facilitate the implementation of targeted interventions and policies designed to reduce inequalities and enhance health outcomes in this region. It is expected that health students, younger students who live with health professionals and have enough money for expenses will have higher health literacy.”

2. Methodology

• Unclear Sampling Method: The sampling method is not described in detail, making it difficult to assess the representativeness of the sample. Provide a more detailed explanation of the sampling process, including any stratification or weighting applied to ensure representativeness.

For a better understanding, we have explained our sampling method. Our sample was selected from higher education institutions in the Alentejo region, specifically in Portalegre, Santarém, Évora and Beja. We identified our target population as students enrolled in higher education institutions in the Alentejo region.

We didn't use any stratification or weighting in our study. However, our sampling method was designed to capture a broad and diverse subset of students. Our aim remained to gain an overall understanding of the student population in the Alentejo region.

No stratification or weighting was applied in our sampling process, as the objectives of our study are specifically centred on this region and its higher education institutions. Our aim is not to generalise the results beyond this context. We don't intend to generalise the results to every student at every institution. The sample has a margin of error of 3%, which is low in most cases and guarantees the robustness of our results.

• Lack of Detail on Data Collection: The data collection process is not fully detailed, including how the survey was administered and how responses were recorded.

Discuss potential biases introduced by the online collection method and steps taken to mitigate these issues.

We have expanded the description of our data collection process, including how the survey was administered online and the steps taken to mitigate potential bias introduced by this method.

The following information has been added:

“Institutional Approval and Promotion

The governing body of each institution was contacted via email to explain the aims and importance of the study, and to request their approval. Once approval was granted, the institution promoted the link to the questionnaire, along with the consent form, to students via their official social media platforms and email lists. To reinforce the importance of participation, and encourage participation, course coordinators were asked to highlight the importance of the survey in emails and meetings. This multi-channel approach was intended to ensure high visibility and maximise response rates.

Survey Administration

Responses were collected electronically using an online survey tool that automatically recorded and securely stored data. The tool ensured anonymity and confidentiality in line with ethical research standards. Regular reminders were sent to students to maximise participation, and technical support was provided to resolve any problems encountered when completing the survey. The survey was designed to be mobile-friendly, ensuring accessibility on various devices, including smartphone or tablets. It was conciseness to minimise the time required for completions.

Addressing Potential Biases

The potential biases inherent in the online data collection method were recognised, including the exclusion of students without internet access, electronic devices or familiarity with digital technologies. To mitigate these concerns, the survey was made accessible on various devices available at higher education institutions, and participants were provided with technical support available at institutions where necessary. In addition, the multi-channel promotion strategy aimed to widen participation across different student populations, thus ensuring a more diverse and representative sample. “

3. Results

Problems:

• Overemphasis on Descriptive Statistics: The results section heavily focuses on descriptive statistics without sufficient emphasis on inferential statistics that address the research questions. More detailed analysis, including regression models and other inferential statistics, is needed to identify determinants of health literacy.

In response to your feedback, we have enhanced the results section with more detailed inferential statistics, such as regression models, to better address the research questions and identify determinants of health literacy.

It was added the following information:

“The multiple linear regression model presented in Table 4 shows the relationship between health literacy and several independent variables among higher education students in the Alentejo region. The independent variables included were age, presence of chronic disease, attendance in health-related courses, living with a health professional, availability of money for expenses, perceived health status, and academic institution. The intercept represents the baseline health literacy score.

The results show that younger students, particularly those aged 16-20 and 21-25, had significantly lower health literacy scores than those aged 26-30, with differences of 3.5 and 3.4 points respectively (p < 0.001). The absence of chronic disease was associated with a higher health literacy score of 1.8 points (p = 0.002), suggesting better health literacy among students without chronic disease. Not attending a health-related course was associated with a significantly lower health literacy score of 9.8 points (p < 0.001), highlighting the impact of health education on health literacy levels.

The data also show that not living with a health professional was associated with a lower health literacy score of 4.2 points (p < 0.001), suggesting that living with health professionals has a positive impact on health literacy. Poor availability of money for expenses was associated with a lower health literacy score by 6.7 points (p < 0.001), highlighting the role of economic factors in health literacy. In addition, perceiving one's health status as unsatisfactory was associated with a lower health literacy score by 4.3 points (p < 0.001), indicating a relationship between self-perceived health and health literacy.

Finally, differences in health literacy were observed between academic institutions were observed. Students from Beja had a higher health literacy score by 2.8 points higher than those from Évora (p < 0.001), students from Portalegre had a score 3.9 points higher (p < 0.001), and students from Santarém had a score 2.0 points higher (p = 0.012).“

• Lack of Contextual Interpretation: Results are presented without sufficient interpretation in the context of the study's aims and existing literature.

Results are now interpreted more robustly within the context of our study aims and the existing literature, ensuring a clearer link between findings and their implications.

4. Discussion

• Overgeneralization: The discussion tends to generalize the findings without acknowledging the specific context of the Alentejo region or higher education students.

We have revised the discussion to avoid over-generalising the findings and to emphasise the specific context of the Alentejo region and and higher education students in our interpretations.

• Lack of Critical Evaluation: There is insufficient critical evaluation of the study's findings, including potential biases, limitations, and implications for future research.

A more critical evaluation of the study results, including potential biases and limitations, has been included to provide a balanced perspective and guide future research directions.

The following information has been added:

“Strengths and Limitations of this Study

A critical evaluation of the study's findings reveals several potential biases, limitations and implications for future research. One potential bias is sampling bias, where the sample, although representative with a margin of error of 3%, may not fully represent the diversity of the higher education student population in the Alentejo region. Although efforts were made to reach a wide audience through multiple dissemination channels, inherent biases in voluntary response rates may persist.

In addition, response bias could be an issue as students who chose to participate may have different characteristics to those who did not, potentially skewing the results. Mitigation efforts included regular reminders and incentives to increase participation to reduce non-response bias. Another concern is the digital divide, where differences in access to digital resources may have affected the participation rates and the representativeness of the sample. Although the survey was designed to be mobile-friendly and institutions provided access to the necessary technology, some inequality may still exist.

The focus of the study on the Alentejo region limits the generalisability of the findings to other regions, highlighting geographical specificity as a limitation. Future studies should consider a broader geographical scope to increase the applicability of the results. In addition, the reliance on self-reported data may introduce social desirability bias.

It is important to recognise that the study had some limitations. The results should be interpreted with caution. The baseline level of health literacy was found to be low, and the study design (cross-sectional) does not allow to establish causal relationships, as only some of the potential determinants/factors associated with the level of health literacy among higher education students in Alentejo were analysed. Nevertheless, it is possible to identify a potential health vulnerability. Previous research has shown that health literacy interventions in higher education can significantly improve students' ability to make informed health-related decisions, thereby reducing their vulnerability to poor health outcomes. (50). The implementation of training programmes focused on healthcare, disease prevention and health promotion could significantly improve the health literacy of university students in Alentejo.

Despite these limitations, the study provides valuable insights into the health literacy of higher education students in Alentejo. By addressing these biases and limitations in future research, we can improve the accuracy and applicability of the findings, ultimately leading to more effective health literacy interventions. “

5. Conclusion

• Overly General Conclusions: The conclusions drawn are too general and do not adequately reflect the specific findings of the study.

o Example: "This study will contribute to the existing literature by elucidating the health literacy level among higher education students in Alentejo, Portugal, and its determinants."

• Lack of Specific Recommendations: The conclusion does not provide specific recommendations for interventions or policy changes based on the findings.

The conclusions have been revised to better reflect the specific findings of our study, to provide a concise summary, and to offer specific recommendations for interventions and policy changes based on our findings.

Recommendations:

• Provide a concise summary of the key findings and their implications.

• Offer specific recommendations for health literacy interventions and policy changes based on the findings.

• Summarize the study's limitations and discuss their implications for future research and practice.

We now provide specific recommendations for health literacy interventions and policy changes based on our findings, ensuring that the conclusions are actionable and relevant to stakeholders.

In summary, these revisions aim to strengthen the manuscript by addressing the specific concerns raised and ensuring clarity, rigour, and relevance throughout. We believe that these changes significantly improve the manuscript and look forward to your further feedback.

The following information has been added:

“The study identified lower levels of health literacy among higher education students in the Alentejo during the 2022/2023 academic year, highlighting significant challenges. Key determinants of health literacy were identified, including age, presence of chronic disease, course type, living with health professionals, financial resources, perceived health status, and academic institution. Younger students, those with chronic conditions, those not enrolled in health-related courses, those not living with a health professional, those with limited financial resources and those who perceived their health as unsatisfactory had lower levels of health literacy. These deficits may affect their wellbeing and future health outcomes. Understanding these determinants is crucial for the development of targeted interventions and educational programmes to improve health literacy and promote better health outcomes among higher education students in the region.

Given their significant social influence, higher education students, play a vital role in shaping health-related decisions for themselves and their communities, particularly in a region facing a growing ageing population that poses challenges to health care and social cohesion. The results show significant differences in health literacy, indicating difficulties in understanding health information and making informed decisions. Comparison of these findings with data from other countries can further inform strategies to improve the health and well-being of university students. Improving socio-economic support, reducing inequalities and preventing student attrition are essential steps in addressing these challenges.

Overall, the study indicates the need for higher education institutions to prioritise the improvement of health literacy. By identifying specific factors influencing health literacy levels in this population, the study enriches the existing literature and provides important information for educators, policy makers and health professionals involved in decision-making processes within higher education. Efforts should focus on promoting all domains of health literacy: health care, disease prevention and health promotion. This study emphasises the need to integrate health literacy into educational curricula and policy frameworks to effectively address these gaps.

Based on the results of the study, targeted health literacy interventions should be implemented to meet the identified needs of higher education students in the Alentejo region. For example, the implementation of interactive workshops on navigating health systems and promoting healthy lifestyles could improve students' understanding and decision-making about their health. In addition, policy changes should prioritise the integration of health literacy into higher education curricula to ensure comprehensive health education.

The limitations of the study highlight the need for caution in interpreting the results. Reliance on self-reported data and potential bias in participant selection may have influenced the results. Future research should use longitudinal approaches to track changes in health literacy over time and assess the effectiveness of different intervention strategies. These findings are essential to refine health literacy interventions and ensure that they meet the diverse needs of higher education students.

In conclusion, the results of this study highlight the urgent need to address the low levels of health literacy among higher education students in the Alentejo region. To mitigate the risks associated with low health literacy, it is essential to implement targeted interventions that foster informed decision-making and promote healthier lifestyles. Educational institutions should consider developing comprehensive curricula that include health literacy as a core competency, as well as offering practical workshops and establishing support networks involving health professionals.

In addition, future research should aim to use mixed methods to gain deeper insights into the dynamics of health literacy, expand geographically to improve comparability, and use longitudinal approaches to track changes over time. By prioritising health literacy in education and public health policy, we can empower the students with the skills necessary to improve health outcomes and ultimately contribute to a healthier society. “

Specific Changes Made:

- Introduction expanded with more context and previous research
- Clearer theoretical framework and hypotheses added
- Methodology section detailed with sampling, data collection, and bias mitigation
- Results section enriched with inferential statistics and contextual interpretation
- Discussion enhanced with comparisons and implications
- Conclusion summarised with key findings and future directions

We hope these revisions meet your expectations and improve the clarity, context, and impact of our manuscript. Thank you again for your valuable insights and recommendations.

Sincerely,

Jorge Rosário

Sara Simões Dias

Sónia Dias

Ana Rita Pedro
